# Methyl 2-(Methylthio)Benzoate: A Sex Attractant for the June Beetles, *Phyllophaga tristis* and *P. apicata*

**DOI:** 10.1673/031.011.10801

**Published:** 2011-08-25

**Authors:** Paul S. Robbins, Glenn A. Salsbury, Robert E. Woodruff, Stephen L. Lapointe, Charles E. Linn

**Affiliations:** ^1^ Department of Entomology, 630 West North St., Cornell University, New York State Agricultural Experiment Station, Geneva, New York 14456; ^2^ Present address: United States Horticultural Research Laboratory, Subtropical Insects Research Unit, ARS, USDA, 2001 South Rock Rd., Fort Pierce, Florida 34945; ^3^ Kansas Department of Agriculture, Plant Protection and Weed Control, Forbes Field Building 282, Box 19282, Topeka, Kansas 66619; ^4^ Florida State Collection of Arthropods, Department of Entomology, P.O. Box 147100, Gainesville, Florida 32614

**Keywords:** *amplicornis*, *crinita*, *Hssopyge*, pheromone, phylogenetics, *suttonana*, *Trichesthes*

## Abstract

Male antennae of *Phyllophaga tristis* (Fabricius) (Coleoptera: Scarabaeidae: Melolonthinae) were tested using a coupled gas chromatograph-electroantennogram detector (GC-EAD) system for electrophysiological responses to five sex pheromones identified from other *Phyllophaga* species including L-valine methyl ester, L-isoleucine methyl ester, L-leucine methyl ester, methyl 2(methylthio)benzoate and methyl 2-amino benzoate. Male antennae responded only to methyl 2(methylthio)benzoate. In a 2003 field test near Greensburg, Kansas, cross-vane traps baited with rubber septa containing 1 mg of methyl 2-(methylthio)benzoate captured 466 male *P. tristis*. Control traps baited with rubber septa loaded with only hexane captured none. Similarly, in a field test in 2010 in Gainesville, Florida, 265 male *P. apicata* Reinhard were captured in traps baited with 1 mg of methyl 2-(methylthio)benzoate whereas control traps captured only a single male.

## Resumen

Usando un cromatógrafo de gases acoplado a un sistema detector electroantenograma (GC-EAD), antenas de machos de *Phyllophaga tristis* (Fabricius) (Coleoptera: Scarabaeidae: Melolonthinae) fueron analizadas para determinar las respuestas electrofisiológicas a cinco feromonas sexuales identificadas en otras especies de *Phyllophaga* incluyendo L-valina metil éster, L-isoleucina metil éster, L — leucina metil éster, metil-2 - (metiltio) benzoato y metil 2-amino benzoato. Antenas de machos solo respondieron a metil-2 - (metiltio) benzoato. En 2003, en evaluaciones de campo cerca de Greensburg, Kansas, trampas cebadas con tapones de caucho conteniendo 1 mg de metil-2 - (metiltio) benzoato capturaron 466 machos de *P. tristis*. Trampas cebadas con tapones de caucho conteniendo unicamente hexano no capturaron insectos. De igual forma, en evaluaciones de campo en el 2010 en Gainesville, Florida, 265 machos de *P. apicata* Reinhard fueron capturados en trampas cebas con 1 mg de metil-2 - (metiltio) benzoato mientras que en las trampas control unicamente se capturo un solo macho.

## Introduction

*Phyllophaga tristis* (Fabricius) and *P. apicata* Reinhard (Coleoptera: Scarabaeidae: Melolonthinae) are two of 861 extant species in the genus *Phyllophaga* (*s. lato*) found in the New World (Evans and Smith 2009). Reinhard (1939) described four subspecies from the *P. tristis* group. Those four subspecies included *P. tristis tristis* (the original species described by Fabricius in 1781) as well as the new subspecies *P. tristis apicata*, *P. tristis amplicornis* and *P. tristis suttonana*. Sanderson (1944) recognized *P. tristis apicata* as the species *P. apicata* Reinhard and returned *P. tristis tristis* to full species status. Later workers (Woodruff and Beck 1989; Riley and Wolfe 2003) also recognized *P. tristis* and *P. apicata* as different species. Additionally, Riley and Wolfe (2003) considered all four as distinct species. *P. amplicornis* Reinhard is endemic to Texas and *P. suttonana* Reinhard nearly so as it may also occur in northeastern Mexico (Riley and Wolfe 2003). *P. apicata* is distributed throughout the southeastern United States from Maryland, Washington D.C. and North Carolina south to Florida and west to Kansas and Texas (Woodruff and Beck 1989; Harpootlian 2001). *P. tristis* is found in Canada and throughout the eastern part of the United States from Maine to Florida and west to North Dakota, Nebraska, Colorado, Arizona, New Mexico and Texas, as well as in Mexico (Luginbill and Painter 1953; Pike et al. 1977; Woodruff and Beck 1989; Harpootlian 2001; Evans and Smith 2009). Drawings of the genitalia of *P. tristis* and *P. apicata* can be seen in Harpootlian (2001) and scanning electron microscope images in Woodruff and Beck (1989). Reinhard (1941) published the only known biological data comparing *P. tristis*, *P. amplicornis*, and *P. apicata*. He enumerated the number of eggs laid by females, the duration of the egg, larval and pupal stages for each of the species and also noted the host plants upon which adults were found feeding.

*P. tristis* was chosen for investigation because this species had been recently removed from the genus *Phyllophaga* (*s. stricto*) to the resurrected melolonthine genus *Trichesthes* (Coca-Abia 2002) and it was anticipated that identification of the sex pheromone might provide useful information for future phylogenetic analyses. The original purpose of this study was to determine the sex pheromone of *P. tristis*. Instead, a sex attractant for *P. tristis* was identified. Additionally, the same compound was found to be a sex attractant for *P. apicata*. Under common usage, a sex pheromone is defined as a compound or compounds identified from a conspecific that elicits a behavioral response in the opposite sex, whereas a sex attractant also elicits a behavioral response, but was not identified from a conspecific of the opposite sex. Foster and Harris (1997) used the word sex attractant (as opposed to sex pheromone) to describe a compound that, while an effective bait for capturing male moths in traps, had not been identified from conspecific females.

## Materials and Methods

### P. tristis

Male *P. tristis* were collected by net in early April 2003 near Greensburg, Kansas. Several attempts were made (netting, light trapping, digging in nearby soil) to collect females for volatile collection, but none were found. Males were packaged individually in one oz cups containing field soil for overnight shipment to Geneva, New York.

Using a coupled gas chromatographelectroantennogram detector (GC-EAD) system, six male antennae (from six different males) of *P. tristis* were tested for electrophysiological responses to five sex pheromones identified previously from other *Phyllophaga* species. These pheromones included the methyl esters of L-valine and L-isoleucine identified from *P. anxia* (Zhang et al. 1997), L-leucine methyl ester identified from *P. (Tostegoptera) lanceolata* (Nojima et al. 2003), methyl 2-(methylthio)benzoate identified from *P. crinita* (Robbins et al. 2003) and methyl 2-amino benzoate identified from *P. (Listrochelus) fimbripes* (unpub. data P.S.R). Neat compounds were diluted in hexane to yield the 20 ng/µl solutions that were used for injection. The GC-EAD instrumentation employed was as described in Robbins et al. (2003).

The electrophysiological response of male *P. tristis* antennae only to the methyl 2(methylthio)benzoate suggested that a field trial was warranted to test for a behavioral response.

On 27 April 2003, a field test was deployed near Greensburg, Kansas at the location where the *P. tristis* males had been collected. The test consisted of three replications of two traps each. Each replication consisted of a blank (control) trap and a test trap. Each control trap was baited with a red rubber septum (Thomas Scientific, www.thomassci.com/index.jsp) containing 50 µl of hexane, whereas each test trap was baited with a septum containing 1 mg of methyl 2-(methylthio)benzoate (Lancaster Synthesis, www.alfa.com/). The lures containing methyl 2-(methylthio)benzoate were made by dissolving the neat compound in hexane to yield a 20 µg/µl solution and dispensing 50 µl of this solution into each septum. The hexane was allowed to evaporate in a fume hood. Septa were placed in labconstructed cross vane traps (see Robbins et al. 2006 for a description and photo). Traps were set in a randomized design ca. 15 m apart such that the trap bottom was ca. 0.5 m from the ground. Traps were checked daily for a total of seven times from 27 April to 4 May 2003. Traps were re-randomized on 2 May.

### P. apicata

The 2010 field test in Gainesville was initiated because of the positive response of *P. tristis* males to methyl 2-(methylthio)benzoate. We hypothesized that *P. apicata* males might also respond because of the close relationship between these species.

On 19 March 2010, a field test was deployed in Gainesville, Florida at a location where *P. apicata* individuals had been collected in previous years. Trapping protocol and baits were as described above for *P. tristis.* Traps were checked daily until flights ended on 17 April. Lures were changed on 4 April and 16 and traps were re-randomized on 26 March and 1, 9, and 16 April.

### Statistical anlaysis

The catch data were tested for homogeneity of variance using Levene's test. No transformations were required. Data were analyzed using a two-sample *t* test, α=0.05.

### Results

Six antennae from six *P. tristis* males responded to methyl 2-(methylthio)benzoate, but not to L-valine methyl ester, L-isoleucine methyl ester, L-leucine methyl ester or methyl 2-amino benzoate. Thus, only methyl 2(methylthio)benzoate was deployed in the field to test for male capture. Electrophysiological responses of the antennae of *P. tristis* males mirrored those seen from *P. crinita* (Burmeister) male antennae when tested with methyl 2(methylthio)benzoate (Robbins et al. 2003).

**Figure 1.**
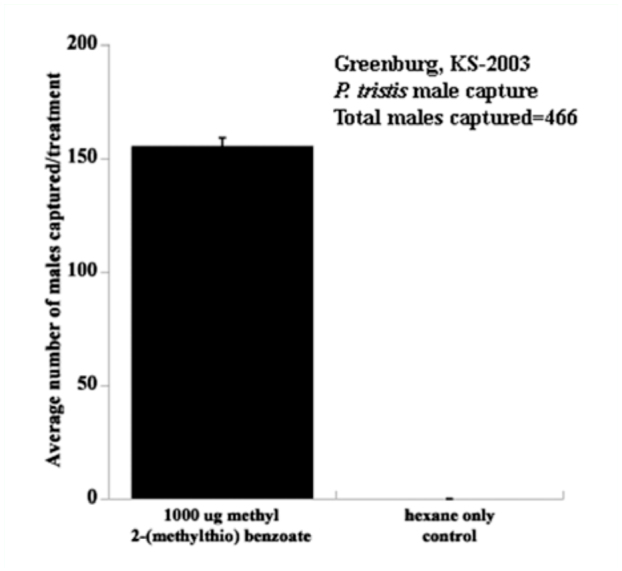
Mean number (± SE, n=3) of male *Phyllophaga tristis* captured in each treatment. Treatments are significantly different using a two-sample *t* test [t (4) = 40.41 ; *P* <0.0001]. High quality figures are available online

In Greensburg, Kansas in 2003, the traps baited with septa containing 1 mg of methyl 2-(methylthio)benzoate captured significantly more *P. tristis* males compared with traps baited with septa containing only hexane [*t* (4) = 40.41; *p* <0.0001, Figure 1]. A total of 466 male *P. tristis* (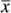 = 155.3, ± 3.84 SE, n=3) were captured between 27 April and 2 May. No males were captured in the hexane only control traps. Captured males were verified as *P. tristis* by comparing them with specimens of P. *tristis* and *P. apicata* supplied to G.A.S by Edward G. Riley of Texas A & M University, College Station, Texas.

**Figure 2.**
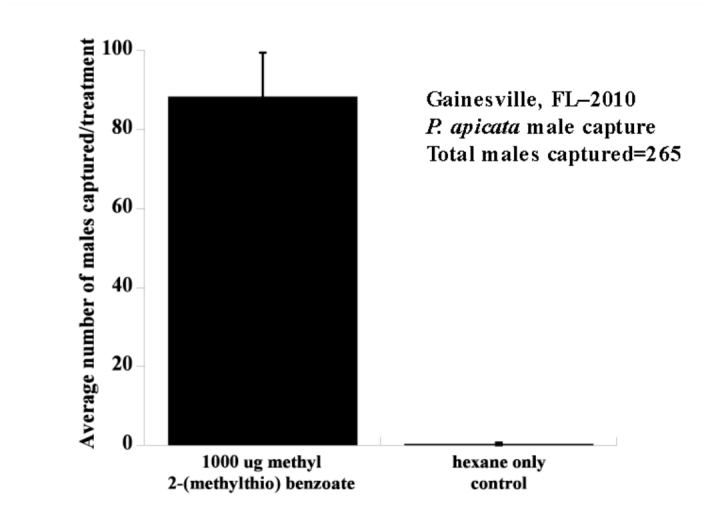
Mean number ((± SE, n=3) of male *Phyllophaga apicata* captured in each treatment. Treatments are significantly different using a two-sample *t* test [*t* (4) = 7.90; *P* = 0.0014]. High quality figures are available online

**Figure 3.**
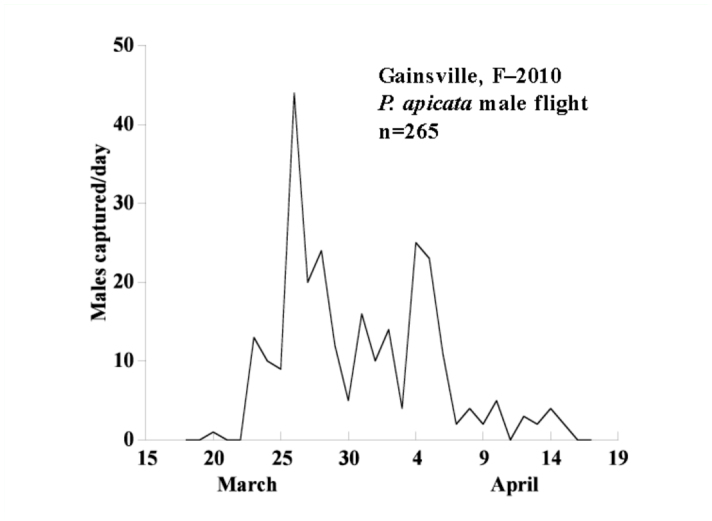
*Phyllophaga apicata* flight data, Gainesville, FL, 2010. High quality figures are available online

In Gainesville, Florida in 2010, the traps baited with septa containing 1 mg of methyl 2-(methylthio)benzoate captured 265 male *P. apicata* (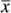
= 51.8, ± 11.14 SE, n=3) whereas the traps baited with septa containing only hexane captured one. [*t* (4) = 7.90; *p*= 0.0014, Figure 2]. These males represent the entire flight period (Mar 20-Apr 16, Figure 3). Captured males were verified as *P. apicata* by R.E.W.

## Discussion

Our results demonstrate that methyl 2(methylthio)benzoate is a sex attractant for both *P. tristis* and *P. apicata*. Analysis of the female-produced volatiles of these species and the proper identification of their sex pheromones remains to be accomplished. Methyl 2-(methylthio)benzoate is inexpensive and could be used to determine presence/absence of these species as well as geographical range limits and yearly flight periodicity. It would be useful to investigate whether male *P. amplicornis* and *P. suttonana* are also captured in traps baited with methyl 2-(methylthio)benzoate.

Nothing is known regarding the intraspecific mating behaviors of *P. tristis* and *P. apicata* beyond this report that methyl 2(methylthio)benzoate functions as a male sex attractant for both species. Woodruff and Beck (1989) indicated that the *P. tristis* complex has a large geographic range, the external characters exhibit a great deal of variation and that the various species can only be distinguished by the internal male aedeagus. Presently, the females are inseparable. In view of this, they called for an examination of the *tristis* complex throughout its entire range as well as an examination of the Fabrician type. Although the members of the *tristis* complex appear to be good morphological species, investigations into their mating behaviors in areas of sympatry could provide information about their status as biological species. Molecular techniques could determine whether gene exchange is occurring.

*P. tristis* and *P. apicata* are the third and fourth species recorded in the literature as being captured in traps baited with methyl 2(methylthio)benzoate, the others being *P. crinita* (Robbins et al. 2003) and *P. lissopyge* (Bates) (Morales-Rodriguez et al. 2011). These four species (along with 36 others) were recently remanded to the resurrected genus *Trichesthes* (Coca-Abia 2002). That study, using morphological characters, hypothesized the monophyly of *Trichesthes* as a group independent of and sister to the *Phyllophaga* (*s.str*.). Further studies are needed to clarify this analysis and the taxonomic status of this name (see Evans and Smith 2009).

Roelofs and Brown (1982) was one of the first studies to identify pheromones as useful characters in phylogenetic hypotheses. They noted that since reproductive isolating mechanisms such as sex pheromones are important in speciation, they could be useful as comparative characters in elucidating phylogenetic relationships. Robbins et al. (2006) reported the capture of more than 56,000 male *Phyllophaga* of 61 species in cross-vane traps baited with various blends (100/0, 90/10, 80/20, 60/40, 40/60, 20/80, 10/90, 0/100) of the methyl esters of L-valine and L-isoleucine, the two components of the sex pheromone of *P. anxia* (Zhang et al. 1997). That study identified species-specific blends that function as sex attractants for a large number of species in the genus *Phyllophaga*.

The present study adds to a growing information base about *Phyllophaga* sex pheromones and sex attractants. This information will aid in elucidating phylogenetic relationships within and between the often confusing and long-disputed *Phyllophaga* groups.
